# Supplementary material for: Beyond individual choice: Exploring pathways linking couple dynamics with Unintended Pregnancy and Birth in India
Source: PLoS One. 2026 Mar 23;21(3):e0344370. doi: 10.1371/journal.pone.0344370 (PMC13008048; doi:10.1371/journal.pone.0344370)
Supplement: S1 Table — (DOCX) [file pone.0344370.s001.docx]

**S1 Table. Pattern of unintended pregnancy and unintended birth across background characteristics.**

| **Characteristics** |  | Unintended Current pregnancy | P value | Unintended Last Birth | P value |
| --- | --- | --- | --- | --- | --- |
|  |  | 11% (weighted) |  | 7.64 % (weighted) |  |
| **Wife Age group** | 15-24 | 9.5 | 0.01 | 6.7 | <0.001 |
|  | 25-34 | 11.9 |  | 7.4 |  |
|  | 35 -49 | 22.2 |  | 12.5 |  |
| **Husband Age Group** | 15-24 | 9.2 | 0.01 | 6.6 | <0.001 |
|  | 25-34 | 10.5 |  | 7.1 |  |
|  | 35-44 | 14 |  | 8.5 |  |
|  | 45 -54 | 31.7 |  | 11.7 |  |
| **Wife’s level of education** | no education | 15.8 | <0.001 | 9.6 | <0.001 |
|  | primary | 17.3 |  | 9.1 |  |
|  | secondary | 9.8 |  | 7.2 |  |
|  | higher | 5.6 |  | 5.9 |  |
| **Husband's educational level** | no education | 15.3 | <0.001 | 10.3 | <0.001 |
|  | primary | 18.6 |  | 8.8 |  |
|  | secondary | 10.2 |  | 7.3 |  |
|  | higher | 6 |  | 6.2 |  |
| **Wife's Occupation** | not working | 12 | 0.01 | 7.6 | 0.04 |
|  | salaried | 1.8 |  | 7.9 |  |
|  | agricultural | 9.6 |  | 6.8 |  |
|  | skilled & unskilled manual | 8.7 |  | 8.9 |  |
| **Husband's Occupation** | not working | 4.6 | 0.13 | 8.5 | 0.4 |
|  | salaried | 9.7 |  | 6.9 |  |
|  | agricultural | 12.8 |  | 7.8 |  |
|  | skilled & unskilled manual | 11.1 |  | 8 |  |
| **Mass media exposure for contraceptives (wife)** | no | 12.8 | 0.08 | 8.7 | 0.01 |
|  | yes | 10 |  | 7.1 |  |
| **Mass media exposure for contraceptives (husband)** | no | 13.3 | 0.05 | 8.2 | 0.2 |
|  | yes | 9.9 |  | 7.4 |  |
| **Household Wealth** | Poorest  Poorer | 34.03  23.44 | <0.001 | 26.77  20.59 | <0.001 |
|  | Middle | 16.36 |  | 19.88 |  |
|  | Richer | 15.60 |  | 18.3 |  |
|  | Richest | 10.58 |  | 14.45 |  |
| **Wife's Religion** | Hindu | 10.8 | 0.08 | 7.6 | 0.8 |
|  | Muslim | 13.8 |  | 8.1 |  |
|  | Christian | 9.4 |  | 7.6 |  |
|  | others | 0.2 |  | 8.2 |  |
| **Husband's Religion** | Hindu | 11.1 | 0.1 | 7.6 | 0.9 |
|  | Muslim | 12.4 |  | 8.1 |  |
|  | Christian | 6.2 |  | 7 |  |
|  | others | 4.7 |  | 7.4 |  |
| **Wife's Caste** | SC | 14.4 | 0.1 | 7.8 | 0.7 |
|  | ST | 9.3 |  | 6.8 |  |
|  | OBC | 9.8 |  | 7.7 |  |
|  | Others | 10.8 |  | 7.8 |  |
| **Husband's Caste** | SC | 13.7 | 0.2 | 8.5 | 0.15 |
|  | ST | 9.4 |  | 6.2 |  |
|  | OBC | 10.4 |  | 7.5 |  |
|  | Others | 10 |  | 7.6 |  |
| **Place of Residence** | rural | 11.8 | 0.12 | 8 | 0.09 |
|  | urban | 8.8 |  | 6.8 |  |
| **Years of marriage** | upto5years | 7.6 | <0.001 | 5.5 | <0.001 |
|  | 6-10years | 10.9 |  | 6.8 |  |
|  | 11to15years | 17.2 |  | 8.2 |  |
|  | 16to25 years | 30.2 |  | 13.4 |  |
|  | >25years | 12.9 |  | 15.1 |  |
| **Number of living Children** | no child | 4.3 | <0.001 | 0.7 | <0.001 |
|  | 1 or 2 children | 13.4 |  | 5.3 |  |
|  | 3 or more children | 27.1 |  | 14.1 |  |
| **Husband's Sexual attitude** | Progressive | 10.76 | 0.21 | 7.3 | 0.23 |
|  | Regressive | 11.35 |  | 8.1 |  |
| **Wife's health Autonomy** | no | 15 | <0.01 | 9.2 | <0.01 |
|  | yes | 9.8 |  | 7.2 |  |
| **Violence** | no | 9.29 | <0.001 | 7.1 | <0.001 |
|  | yes | 19.19 |  | 9.5 |  |
