# Supplementary material for: Beyond individual choice: Exploring pathways linking couple dynamics with Unintended Pregnancy and Birth in India
Source: PLoS One. 2026 Mar 23;21(3):e0344370. doi: 10.1371/journal.pone.0344370 (PMC13008048; doi:10.1371/journal.pone.0344370)
Supplement: Attachment — Submitted filename: Response to Reviewers.docx [file pone.0344370.s002.docx]

**Response to Editor and Reviewers-**

We sincerely thank the Editor and the reviewers for their careful reading of the manuscript and for their constructive and insightful comments. The suggestions have substantially helped improve the clarity, rigor, and overall quality of the paper. All comments have been carefully considered, and the manuscript has been revised accordingly. Our point-by-point responses are provided below.

**Journal Requirements:**

1. Please ensure that your manuscript meets PLOS ONE's style requirements, including those for file naming.

**Response from Author –** All necessary changes have been made to ensure that the manuscript complies with PLOS ONE’s style requirements, including file naming.

2. When completing the data availability statement of the submission form, you indicated that you will make your data available on acceptance. We strongly recommend all authors decide on a data sharing plan before acceptance, as the process can be lengthy and hold up publication timelines. Please note that, though access restrictions are acceptable now, your entire data will need to be made freely accessible if your manuscript is accepted for publication. This policy applies to all data except where public deposition would breach compliance with the protocol approved by your research ethics board. If you are unable to adhere to our open data policy, please kindly revise your statement to explain your reasoning and we will seek the editor's input on an exemption. Please be assured that, once you have provided your new statement, the assessment of your exemption will not hold up the peer review process.

**Response from Author –** The source of the data has now been clearly specified in the Data Availability Statement.

3. We note that Figure 2 in your submission contains copyrighted images. All PLOS content is published under the Creative Commons Attribution License (CC BY 4.0), which means that the manuscript, images, and Supporting Information files will be freely available online, and any third party is permitted to access, download, copy, distribute, and use these materials in any way, even commercially, with proper attribution.

**Response from Author –** Figure 2 was prepared by the author using analysed data and does not include copyrighted material. As the analysis has been revised, the earlier figure has been replaced with a newly prepared figure, which is also entirely created by the author.

**Comments of Editor**

The author is suggested to revise the paper on the following four aspects-

1. Operational Definition of Unintended Pregnancy-

**Response from Author** – **The** outcome variable has been revised and is now termed Unintended Pregnancy and Birth (UPB). The revised manuscript clearly explains the construction of this variable.

2. Theoretical framework and conceptualization on pathways

**Author’s Response -** The theoretical framework has been restructured by synthesizing relevant theories more explicitly, and the conceptual framework has been revised accordingly to clarify the proposed pathways.

3.  Inclusion/exclusion variables in different indices used in the study

**Response from Author –** The construction of all indices has now been described in detail.

4. The model used to measure the risk of process/outcome. Author may consider a separate model on current pregnancy intendedness, last birth intendedness and abortion related reasons depicting unintendedness. The final model may be developed based on the 3 components if considered as an unintended pregnancy

**Response from Author –** Following the Editor’s suggestion, the outcome variable has been revised to Unintended Pregnancy and Birth (UPB), combining current unintended pregnancies and unintended last births within five years preceding NFHS-5. Abortion-related unintendedness has been excluded.

Because the analysis is based on the couple file, the sample size for the current pregnancy alone was small, leading to sparse cell issues. To ensure robustness, we conducted separate bivariate analyses for the two components- current unintended pregnancy and unintended last birth, using the Rao–Scott chi-square test. These results are provided as Supplementary Information (S1 Table). The limitation related to post-factum rationalisation of unintended births has been explicitly acknowledged in the revised manuscript.

As the analysis has been substantially revised, the manuscript has undergone thorough editing.

**Comments of Reviewer 1:**

**1.** The theoretical section is rich but somewhat dense, and the explicit hypotheses are not clearly stated. Please add a short subsection at the end of the theoretical framework that explicitly lists the main hypotheses (e.g., H1–H4 on autonomy, attitudes, IPV, mediation, and moderation) to guide the reader and align with the analytic strategy.

**Response from Author-** At the end of the theoretical framework, all the hypotheses are added.

2. The outcome variable “unintended pregnancy” combines current pregnancies, recent births, and abortions due to several reasons (e.g., contraceptive failure, economic constraints). Reporting “wanted later” and “wanted no more” for delivered births may be affected by post-factum rationalisation.

**Response from Author –** The outcome variable has been revised to Unintended Pregnancy and Birth (UPB), abortion-related components have been removed, and the limitation of post-factum rationalisation has been explicitly discussed in the Limitations section.

3. In Table 3 and Figure 1, the direction of the associations between variables is not explicitly indicated. The authors may consider revising these to make the tables and figures more self-explanatory.

**Response from Author –** We used arrows to show the pathway linkages, and the association is described through beta coefficients (β )

4. The authors may provide more detailed justification for the categorisation of the independent variables capturing couple dynamics.

**Response from Author –** Incorporated into revised manuscript.

5.The cross-sectional nature of NFHS-5 limits causal inference, yet some parts of the Discussion use language that may be interpreted as causal. The authors may add a brief paragraph acknowledging that mediation and moderation are modelled conceptually and that temporality cannot be established due to the cross-sectional design.

**Response from Author –** The limitations of the cross-sectional design have been clearly acknowledged, and the Discussion has been revised to avoid causal interpretations. Mediation and moderation are now explicitly framed as conceptual and associative.

6. In Table 3, p-values should not be reported as 0; these should be replaced with “<0.001” where appropriate.

**Response from Author –** P-values are no longer reported as zero. Instead, levels of statistical significance are indicated using *** p<0.01, ** p<0.05, and * p<0.10.

7. The Introduction and Discussion could be slightly streamlined to reduce repetition and to break very long paragraphs into shorter, more focused ones.

**Response from Author –** The Introduction and Discussion have been streamlined, with long paragraphs broken into shorter, more focused sections to reduce repetition.

**Comments of Reviewer 2:**

1. In the methodology section under Data Source, revise the sentence to state that NFHS-5 is a large-scale survey.

**Response from Author –** It is mentioned in the data source (line number- 153)

2. Maintain consistency by reporting numerical values up to one decimal place throughout the manuscript, including all tables.

**Response from Author –** Numerical values are consistently reported up to two decimal places throughout the manuscript, including all tables, to improve precision of confidence intervals.

3. In Table 1, the percentage of unwanted pregnancy is reported as weighted; however, it is not clear whether the total number of pregnancies (sample size) is weighted or unweighted. Please clarify this in the table or its footnote.

**Response from Author –** It is now clearly stated in Table 1 that percentages are weighted, while the sample size is unweighted.

4. In Table 3, reporting both p-values and significance stars (***) is redundant. Please retain either the p-value or the significance stars, and ensure consistency in both the table and the text. If p-values are retained, report them up to three decimal places throughout the manuscript.

**Response from Author –** P-values have been removed, and statistical significance is consistently reported using significance stars only.

5. Although limitations are discussed within the manuscript, please present Limitations and Strengths as a separate, clearly defined section.

**Response from Author –** The section is added.

6. Present the Conclusion as a separate section with an appropriate heading.

**Response from Author –** A separate section for the conclusion has been added.

7. Include Policy Implications and Future Research Directions based on the key findings of the study, if applicable.

**Response from Author –** We thank the reviewer for this suggestion. Rather than adding separate sections, policy implications have been integrated within the Discussion and Conclusion, where they are directly linked to the study’s key findings.
